# HIV disease burden, cost, and length of stay in Portuguese hospitals from 2000 to 2010: a cross-sectional study

**DOI:** 10.1186/s12913-015-0801-8

**Published:** 2015-04-08

**Authors:** Emanuel Catumbela, Alberto Freitas, Fernando Lopes, Maria del Carmen Torres Mendoza, Carlos Costa, António Sarmento, Altamiro da Costa-Pereira

**Affiliations:** Department of Health Information and Decision Sciences, Faculty of Medicine, University of Porto, Porto, Portugal; Department of Pathology, Faculty of Medicine, Universidade Agostinho Neto, Luanda, Angola; CINTESIS–Center for Research in Health Technologies and Information Systems, University of Porto, Porto, Portugal; Division of Global HIV/AIDS, U.S. Centers for Disease Control and Prevention (CDC), Atlanta, USA; National School of Public Health, University Nova de Lisbon, Lisbon, Portugal; Department of Infectious Diseases, Faculty of Medicine, University of Porto, Porto, Portugal; Departamento de Ciências da Informação e da Decisão em Saúde, Faculdade de Medicina, Universidade do Porto, Al. Prof. Hernâni Monteiro, Porto, 4200-319 Portugal

**Keywords:** Advanced HIV infection, Administrative data, HIV epidemiology

## Abstract

**Background:**

The number of HIV-related hospitalizations has decreased worldwide in recent years owing to the availability of highly active antiretroviral therapy. However, the change in HIV-related hospitalizations in Portugal has not been studied. Using comprehensive hospital discharge data from mainland Portuguese hospitals, we examined trends in HIV-related inpatient admissions, length of stay (LOS), Elixhauser comorbidity measures, in-hospital mortality, and mean cost from 2000 to 2010.

**Methods:**

The hospital administrative data from inpatient admissions and discharges at 75 public acute care hospitals in the Portuguese National Health Service from 2000 to 2010 were included. HIV-related admissions were identified using the International Classification of Diseases, 9^th^ Revision, Clinical Modification diagnosis codes 042.x–044.x. The effect of Elixhauser comorbidity measures on extending the LOS was assessed by comparing admissions in HIV patients with and without comorbidities using the Mann–Whitney U test. Multivariate logistic regression was performed to estimate the odds of having a decreased discharge.

**Results:**

A total of 57,027 hospital admissions were analyzed; 73% of patients were male, and the mean age was 39 years. The median LOS was 11 days, and the in-hospital mortality was 14%. The mean cost per hospitalization was 5,148.7€. A total of 83% of admissions were through the emergency room. During the period, inpatient HIV admissions decreased by 22%, LOS decreased by 9%, and in-hospital mortality dropped by 12%. Elixhauser comorbidities increased the median LOS in nearly all admissions.

**Conclusions:**

Despite small regional variations, a strong, consistent decrease was observed in the hospital admission rate, mean cost, length of stay, and mortality rate for HIV-related admissions in Portugal during 2000–2010.

**Electronic supplementary material:**

The online version of this article (doi:10.1186/s12913-015-0801-8) contains supplementary material, which is available to authorized users.

## Background

Since the HIV epidemic emerged in Portugal in 1983, 39,347 cases have been reported, and as of 2010, 16,370 patients have developed advanced HIV infection [1]. Although the incidence of advanced HIV infection is declining worldwide, the population of persons living with HIV (PLWHIV) continues to rise [2]. Studies show that the number of HIV-related hospitalizations has decreased in recent years primarily because of the availability of highly active antiretroviral therapy (HAART) [3-6]. This decrease has been accompanied by an increase in the population of PLWHIV worldwide, which was estimated at 34 million people (31.6–35.2 million) through 2010, a 17% increase since 2001 [7]. HIV-infected individuals are surviving and experiencing longer life expectancies, as well as age-associated comorbidities [8]. Accordingly, the number of hospitalizations has emerged as an important outcome measure and contributor to healthcare expenditures in this population.

To determine the relationship between non-AIDS comorbidities and the progression of HIV disease, in the present study we employed the Elixhauser Comorbidity Index (ECI), which is a comorbidity classification system used to describe and compare patient populations. The ECI can adjust for confounding effects caused by the potential chronic disease burden when a condition is associated with a particular outcome [9]. In a previous study, we found that the ECI was associated with substantial increases in the frequency and duration of hospitalization, and mortality [10]. The ECI evaluates 30 comorbidities including HIV; however, we applied the ECI without including HIV as a comorbidity.

The impact on the Portuguese population of the reduction in HIV/AIDS-related morbidity and hospitalization in the HAART era has not been thoroughly characterized, and only one known study, which primarily examines mortality, has been published [11]. An understanding of the factors affecting HIV-related hospitalizations would help optimize resource allocation among regions and could potentially reduce the hospitalization rate for PLWHIV.

The Portuguese health system comprises three coexisting systems: the National Health Service (NHS), special social health insurance programs for certain professions (health subsystems), and elective private health insurance. The NHS establishes the right of all citizens to health protection; all citizens have a guaranteed universal right to health care through the NHS. The patient pays a specific fixed cost per use for certain health care services delivered by NHS facilities, but HIV drugs are provided free of charge to all HIV patients [12,13].

Using comprehensive hospital discharge data from Portuguese hospitals, we examined the trends in HIV-related inpatient admissions, length of stay (LOS), mean costs, ECI, and HIV in-hospital mortality from 2000 to 2010.

## Methods

This cross-sectional study examined trends in HIV-related inpatient admissions in Portugal. Hospital administrative data were collected from public acute care hospitals in the Portuguese NHS database maintained by the Central Health System Administration (ACSS). Anonymity was maintained for all hospitals and patients. The dataset included 10,586,118 discharges across 75 hospitals in mainland Portugal from January 2000 to December 2010. The dataset also included the principal diagnosis, defined as the principal condition causing the patient’s admission to the hospital, and up to 19 additional diagnoses.

To capture all of the potential HIV hospital admissions, eligible cases met one of the following criteria: (1) principal diagnosis of HIV/AIDS as defined by the International Classification of Diseases, Ninth Revision, Clinical Modification (ICD-9-CM) with diagnosis codes of 042.x–044.x; (2) principal diagnosis of advanced HIV infection (i.e., pneumonia or opportunistic infection as defined by the Major Diagnostic Category 25, “Human Immunodeficiency Virus Infection” [14-16] and a secondary diagnosis of HIV/AIDS; or (3) principal diagnosis unrelated to HIV/AIDS and a secondary diagnosis of HIV/AIDS. Comorbidities were defined as diagnoses other than HIV/AIDS as defined above.

**Figure 1:**
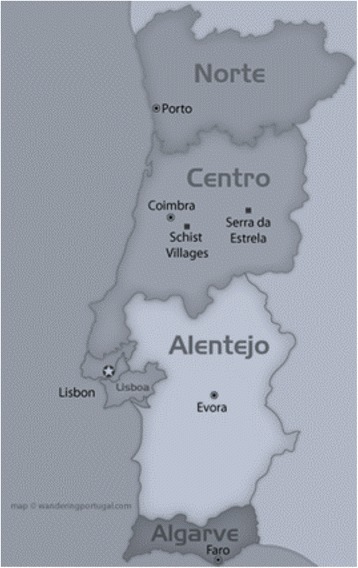
**Portugal** ***Nomenclature of Territorial Units for Statistics*** **II (NUTS II) regional map.** The Norte, Centro, Lisbon, Alentejo, and Algarve regions were included in the present study.

The country-wide distribution of hospitals was determined using the *Nomenclature of Territorial Units for Statistics II* (NUTS II), which is a geocode standard developed by the European Union for referencing the administrative divisions of countries for statistical purposes. The NUTS II level identifies the region by a two-digit or letter-digit code [17] and is only applied to the mainland regions of Portugal, namely Norte, Centro, Lisbon, Alentejo, and Algarve (Figure 1).

From 2000 to 2010, five administrative regulations were issued by the Ministry of Health to assign and update the costs for inpatient admissions according to diagnosis-related groups (DRGs). Two DRGs were used during this period. The mean cost (euros) per hospitalization and the mean yearly cost for each HIV inpatient admission were calculated using both the regulations and discharge DRGs as follows: for the year 2000, regulation 348-B/1998; for 2001–2002, regulation 189/2001; for 2003–2005, regulation 132/2003; for 2006–2008, regulation 567/2006; and for 2009–2010, regulation 132/2009. The DRG 488–490 was used from 2000–2005, and the DRG 700–716 was used from 2006–2010 (Table 1).

**Table 1:** **Administrative regulations from the Portuguese Ministry of Health assigning hospitalization costs according to the DRG**

				**Ordinances**			
**MCD** ^*^	**DRG** ^**^	**Designation**	**348-B/1998**	**189/2001**	**132/2003**	**567/2006**	**132/2009**
**25 HIV infection**	488	HIV with an extensive procedure in the operating room.	€ 2,193.50	€ 11,889.72	€ 13,099.08		
	489	HIV with a significant related clinical situation.	€ 1,121.30	€ 7,181.14	€ 7,911.56		
	490	HIV with procedures in the operating room without other related situations.	€ 664.80	€ 3,007.10	€ 33,212.97		
**24 HIV infection**	700	Tracheostomy for HIV.				€ 39,162.25	€ 40,062.90
	701	Infection with HIV and an operation and ventilation or nutritional support.				€ 14,467.48	€ 14,800.20
	702	HIV infection with an operation room procedure and multiple related major infections.				€ 11,771.16	€ 12,041.88
	703	HIV infection with a block operative procedure and related major diagnosis.				€ 10,433.90	€ 10,673.86
	704	HIV infection with an operating room procedure with no related major diagnosis.				€ 6,235.18	€ 6,378.58
	705	HIV infection with multiple related major infections, including tuberculosis.				€ 8,737.55	€ 8,938.49
	706	HIV infection with multiple related major infections, not including tuberculosis.				€ 8,666.81	€ 8,866.13
	707	HIV infection with a nutritional support or ventilator.				€ 8,421.56	€ 8,615.24
	708	HIV infection with a related major diagnosis, discharge against medical advice.				€ 3,597.43	€ 3,680.16
	709	HIV infection with multiple related major diagnoses or significant diagnoses, including tuberculosis.				€ 8,322.24	€ 8,513.64
	710	HIV infection with multiple related major diagnoses or significant diagnoses, not including tuberculosis.				€ 5,896.94	€ 6,032.56
	711	HIV infection with a major related diagnosis without multiple related major or significant diagnoses, including tuberculosis.				€ 5,030.96	€ 5,146.67
	712	HIV infection with a major related diagnosis without multiple related major or significant diagnoses, not including tuberculosis.				€ 3,277.22	€ 3,352.59
	713	HIV infection associated with a significant diagnosis, discharge against medical advice.				€ 1,847.90	€ 1,890.40
	714	HIV infection with significant related diagnosis.				€ 2,980.44	€ 3,048.99
	715	HIV infection with other related diagnoses.				€ 1,725.63	€ 1,765.32
	716	HIV infection with no other related diagnoses.				€ 1,568.69	€ 1,604.77

^*^MCD = Major Category Diagnosis.

^**^DRG = Diagnosis-related group.

The hospitalization rate was calculated from the 2000–2010 data on HIV infection in Portugal published by the Direcção-Geral de Saúde [18]. The mid-year population of PLWHIV was calculated per year by summing the new and old cases, and dividing the result by two.

### Analysis

Variations in the HIV-related hospitalization rates according to clinical characteristics were assessed by univariate analysis and a linear trends test. We first examined the number of HIV-related admissions per year according to the NUTS II region, median LOS, in-hospital mortality, mean cost, admission type, age, and sex. The units of analysis comprised each combination of sex (two categories), age (four categories), NUTS II region (five categories), year (11 categories), LOS, mortality, and mean cost. The results are represented as the frequency, median, and interquartile range, or as the mean and 95% confidence interval (CI), as appropriate. The impact of the ECI on the LOS was determined by comparing admissions in patients with and without comorbidities using the Mann–Whitney U test at a 0.05 level of significance.

Multivariate logistic regression was performed to estimate the odds of having a decreased discharge status according to all HIV-patient and independent variables, including age (first age category served as the standard: 0 to 17 years, 18 to 45 years, 46 to 65 years, and 66 years and older), gender (males compared with females), LOS, and Elixhauser comorbidities (each comorbidity admission was compared with another admission without that comorbidity), with a p-value < 0.05 indicating statistical significance. The variables (comorbidities) included in multivariate logistic regression analysis were selected based on the results of a univariate analysis. Despite the large number of variables (comorbidities), their use is relevant given their influence on hospital outcomes, particularly on mortality, LOS, and the use of hospital resources. In addition, the study population is quite large and includes many deceased patients; therefore, many cases exist for each predictor and independent variable. The statistical analyses were performed using SPSS version 21 (SPSS IBM, New York, USA).

### Ethical standards

This study employed secondary anonymous data and was performed in accordance with the ethical standards established by the 1964 Declaration of Helsinki and its later amendments. The data were used for scientific research purposes under the Medicine Faculty of University of Porto and Central Health System Administration agreement.

## Results

From 2000 to 2010, 57,027 HIV-related hospital admissions occurred in mainland Portugal. The frequency of admissions decreased during the period, from 5,459 episodes in 2000 to 4,254 episodes in 2010. Additionally, 70% of admissions were male patients, and the mean age increased from 35 years in 2000 to 44 years in 2010. A total of 70% of all admissions were patients aged between 18 and 45 years. The median LOS decreased from 12 days in 2000 to 10 days at the end of the decade. During this time, 8,203 deaths were reported, and the mortality rate decreased from 14.9% to 13.1% (Table 2).

**Table 2:** **Population, hospitalization, and mortality trends in HIV-related inpatient admissions in Portugal**

													**Linear regression**	
**Variables**	**Total**	**2000**	**2001**	**2002**	**2003**	**2004**	**2005**	**2006**	**2007**	**2008**	**2009**	**2010**	**slop**	**95% IC**
Mid-year populations of persons living with HIV*		4,466	4,993	5,524	6,010	6,437	6,854	7,213	7,521	7,819	8,052	8,289	382.127	[341.991:422.264]
HIV/AIDS hospitalization frequency	**57,027**	5,459	5,550	5,654	5,628	5,468	5,491	5,214	5,089	4,894	4,326	4,254	−132.118	[−186.756: −77.480]
Hospitalization rate^#^		1.2	1.1	1.0	0.9	0.8	0.8	0.7	0.7	0.6	0.5	0.5	−0.069	[−0.078: −0.060]
Gender (% male)	**73**	76	76	76	74	74	73	74	72	70	70	70	−0.691	[−0.854:−0.528]
Age (mean)	**39**	35	37	38	38	39	41	41	42	42	43	44	0.827	[0.709:0.946]
Median LOS (days)	**11**	12	12	12	11	12	12	11	11	11	10	10	−0.200	[−0.296:−0.104]
In-hospital mortality (%)	**14.4**	14.9	15.5	15.4	15.1	14.9	14.8	13.8	13.4	13.1	13.4	13.1	−0.262	[−0.349:−0.175]
Average cost (€)	**5,148.7**	997.7	4,818.1	6,062.0	6,625.6	6,689.7	6,640.8	5.676.4	4,480.0	4,670.4	4,594.1	4,664.0	72.329	[−293.079:437.737]
Total cost in M (€)	**293,614**	5,446	26,740	34,274	37,288	36,579	36,464	29,596	22,798	22,856	19,874	19,840	−233.736	[−2437.823:1970.350]

*Data published in “Infecção VIH/SIDA: A Situação em Portugal a 31 de Dezembro de 2010,” by the Departamento de Doenças Infecciosas.

^#^Calculated by dividing the mid-year population of persons living with HIV by the number of hospitalization episodes due to HIV per year.

95% CI = 95% confidence interval.

When examined by the NUTS II region (Table 3), admissions were most frequent in Lisbon and Norte, which was expected as these are the most populous areas in Portugal. From 2000 to 2010, the number of inpatient admissions decreased by 35% in Lisbon, 22% in Algarve, and 4% in Norte, but increased by 31% in Centro and approximately 20% in Alentejo. The in-hospital mortality rate in Norte decreased by 40% over the period, from 19.1% in 2000 to 11% in 2010, while in Centro, the mortality decreased by only 24%, dropping from 10% in 2000 to 7.7% in 2010. In Lisbon, Alentejo, and Algarve, the mortality rates increased by 3%, 34%, and 52%, from 14.5%, 17%, and 8.3% in 2000 to 15%, 23%, and 12% in 2010, respectively. The most deaths in the Norte region (19%) occurred in 2000, for Centro (15%) in 2006, for Lisbon (16%) in 2001, for Alentejo (24%) in 2002, and for Algarve (15%) in 2003.

**Table 3:** **Median HIV inpatient admissions in Portugal by NUTS II region and year**

**NUTS II / Year**	**2000**	**2001**	**2002**	**2003**	**2004**	**2005**	**2006**	**2007**	**2008**	**2009**	**2010**	**Total**	**% Change (2000–2010)**
**Norte, N**	**1,191**	**1,274**	**1,356**	**1,298**	**1,327**	**1,473**	**1,384**	**1,374**	**1,294**	**1,208**	**1,139**	**14,318**	**−4.4**
Median LOS	12	11	11	12	13	12	10	11	10	9	9	10.9	−25
% Death	19.1	17.7	17.6	16.4	15.4	15.3	11.7	13.8	12.8	13.5	11.4	15.0	−40.1
**Centro, N**	**415**	**449**	**563**	**552**	**528**	**565**	**539**	**535**	**604**	**536**	**546**	**5,832**	**31.6**
Median LOS	11	11	11	11	11	10	10	11	10	10	9	10.4	−18.2
% Death	10.1	9.4	12.4	8.9	10.8	11.3	14.8	11.2	7.3	7.5	7.7	10.1	−24.0
**Lisbon, N**	**3,511**	**3,412**	**3,319**	**3,366**	**3,237**	**3,020**	**2,908**	**2,712**	**2,609**	**2,226**	**2,272**	**32,592**	**−35.3**
Median LOS	12	12	12	11	12	12	12	11	12	11	11	11.6	−8.3
% Death	14.5	16.1	15.0	15.7	15.3	15.2	14.5	13.8	14.2	14.4	15.0	14.9	3.4
**Alentejo, N**	**76**	**100**	**103**	**132**	**111**	**143**	**137**	**129**	**114**	**98**	**91**	**1,234**	**19.7**
Median LOS	11	10	13	11	12	10	9	9	11	10	9	10.4	−18.2
% Death	17.1	12.0	24.3	15.2	22.5	16.8	17.5	17.1	19.3	19.4	23.1	18.4	34.9
**Algarve, N**	**266**	**315**	**313**	**280**	**265**	**290**	**246**	**339**	**273**	**258**	**206**	**3,051**	**−22.6**
Median LOS	12	12	11	10	11	12	12	11	11	14	13	11.7	8.3
% Death	8.3	10.2	12.1	14.6	12.8	14.1	12.2	11.2	14.3	14.3	12.6	12.4	52.6
**Total, N**	**5,459**	**5,550**	**5,654**	**5,628**	**5,468**	**5,491**	**5,214**	**5,089**	**4,894**	**4,326**	**4,254**	**57,027**	**−22.1**
Median LOS	11.5	11.2	11.6	11.0	11.8	11.2	8.0	10.6	10.8	10.8	10.2	11.0	−8.9
% Death	14.9	15.5	15.4	15.1	14.9	14.8	13.8	13.4	13.1	13.4	13.1	14.4	−11.7

NUTS II = *Nomenclature of Territorial Units for Statistics II*; LOS = length of stay.

The median LOS was 11 days (interquartile range 5–22 days). Between 2000 and 2010, the median LOS across mainland Portugal decreased by 25% (from 12 to 9 days) in the Norte region, 18% (from 11 to 9 days) in the Centro and Alentejo regions, and 8% (from 12 to 11 days) in the Lisbon region, while the median LOS in the Algarve region increased by 8% (from 11 to 13 days). The median LOS was significantly higher in men (12 days; interquartile range 5–23 days) than in women (10 days; interquartile range 2–23 days). This difference was statistically significant (*p* < 0.001) using the Mann–Whitney U test (data not shown).

Hospitalization occurred primarily in two ways: through the emergency room or through an ambulatory consultation. When the data were examined according to the site of admission, we found that 83% of hospitalizations originated from the emergency room. Planned admissions fluctuated substantially during the study period. In 2000, there were 757 planned admissions, which increased to 1,238 admissions in 2004, to 963 admissions in 2005, and to 686 admissions in 2010. The number of admissions from the emergency room increased slightly over the period, from 4,702 admissions in 2000 to 3,573 by 2010 (Additional file 1: Table S1).

The mean cost per HIV admission decreased during the study period and substantially decreased in the Norte, Centro, and Lisbon regions. The mean cost in the Norte region was 5,499€ (95% CI 5,555€–5,444€) over the entire study period, with 2003 and 2004 as the most expensive years (7,137€ and 7,181€; 95% CI 7,006€–7,269€ and 7,058€–7,305€, respectively). In Centro, the overall mean cost was 4,739€ (95% CI 4,822€–4,657€), with 2003, 2004, and 2005 as the most expensive years (6,393€, 6,593€, and 6,474€; 95% CI 6,183€–6,603€, 6,372€–6,814€ and 6,243€–6,704€, respectively). In Lisbon, the mean admission cost was 5,293€, and the most expensive years were 2004 to 2006, with a mean admission cost of over 6,000€ during this period. In Alentejo, the mean admission cost was 5,210€ (5,411€–5,009€). The most expensive years were 2002 to 2005, averaging over 6,000€ each year. In Algarve, the mean admission cost was 4,999€ (95% CI 5,123€–4,875€).

The mean HIV admission cost in mainland Portugal was lowest in 2001 (Table 4). The mean daily cost was 253€ and increased slightly over the decade from 248€ to 260€ (4% increase), while the estimated cost per year decreased considerably from 19 to 15 million euros over the decade (12% decrease). Overall, an estimated 208 million euros were spent on patients with HIV and related diseases. DRGs 710 (21%) and 714 (19%) were the most expensive (Additional file 1: Table S2 and Table S3).

**Table 4:** **Mean costs for HIV inpatients in Portugal by year and NUTS II**

**Year**	**Norte**		**Centro**		**Lisbon**		**Alentejo**		**Algarve**	
	**Mean(€)**	**(95% IC)**	**Mean(€)**	**(95% IC)**	**Mean(€)**	**(95% IC)**	**Mean(€)**	**(95% IC)**	**Mean(€)**	**(95% IC)**
**2000**	1051.0	(1036.8–1065.2)	953.7	(925.2–982.2)	1024.0	(1014.2–1033.8)	982.7	(926.0–1039.4)	977.3	(939.4–1015.2)
**2001**	5099.2	(4928.6–5269.9)	4597.4	(4327.0–4867.8)	4808.6	(4699.8–4917.5)	4961.2	(4324.1–5598.3)	4624.3	(4281.1–4967.4)
**2002**	6480.8	(6376.0–6585.6)	5654.6	(5471.4–5837.7)	6209.4	(6126.9–6291.9)	6356.6	(5986.9–6726.4)	5608.6	(5353.4–5863.8)
**2003**	7137.9	(7006.4–7269.4)	6393.4	(6183.0–6603.9)	6798.8	(6710.4–6887.3)	6370.8	(5911.5–6830.2)	6427.0	(6140.4–6713.7)
**2004**	7181.7	(7058.0–7305.3)	6593.4	(6372.1–6814.7)	6855.2	(6766.0–6944.4)	6418.4	(5944.1–6892.7)	6399.7	(6099.1–6700.3)
**2005**	6894.7	(6772.8–7016.6)	6474.0	(6243.6–6704.4)	6746.2	(6651.4–6840.9)	6459.4	(6061.0–6857.8)	6629.9	(6339.5–6920.4)
**2006**	5924.8	(5777.3–6072.3)	5286.5	(5044.9–5528.1)	6056.5	(5946.7–6166.3)	5601.4	(5096.8–6105.9)	5512.6	(5181.2–5844.1)
**2007**	5110.8	(4936.9–5284.8)	3793.2	(3579.2–4007.2)	4951.4	(4818.9–5083.8)	4335.4	(3541.7–5129.0)	4209.3	(3921.6–4497.0)
**2008**	5301.3	(5103.1–5499.6)	3930.2	(3717.3–4143.1)	5053.6	(4903.7–5203.5)	4403.5	(3849.7–4957.3)	4663.4	(4019.3–5307.5)
**2009**	4907.5	(4705.1–5109.9)	4244.9	(3956.7–4533.1)	4995.3	(4814.3–5176.3)	3898.9	(3375.5–4422.2)	4923.9	(4450.5–5397.3)
**2010**	4733.4	(4553.7–4913.1)	3919.6	(3640.1–4199.1)	4917.4	(4759.6–5075.1)	5169.6	(3923.7–6415.5)	4579.8	(4169.3–4990.4)
**All years**	5499.9	(5555.0–5444.9)	4739.8	(4822.1–4657.6)	5293.2	(5332.5–5.254.0)	5210.9	(5411.8–5009.9)	4999.5	(5123.3–4875.7)

The cost per hospitalization was estimated using the following administrative regulations (named “*Portaria*”) from the Ministry of Health: Portaria 348-B/1998, Portaria 189/2001, Portaria 132/2003, Portaria 567/2006, and Portaria 132/2009. The diagnosis-related groups (DRGs) 488–490 were used from 2000 to 2003, and DRGs 700–716 were used from 2003 to 2010. A total of 25 Major Diagnostic Categories (MDCs) were used from 2000 to 2003, and 24 MDCs were used from 2003 to 2010.

NUTS II = *Nomenclature of Territorial Units for Statistics*; 95% CI = 95% confidence interval.

Based on the ECI, drug abuse was identified in 30% of patients, liver disease in 24%, and fluid and electrolyte disorders in 9%. Nearly every comorbidity significantly increased the LOS. The comorbidities associated with the greatest increase in the median LOS were paralysis (12 days), valvular heart disease (8 days), and hemorrhage (8 days; Table 5).When the Elixhauser comorbidities were examined by gender, because of the predominantly male population, most comorbidities occurred in men (73%). When the number of comorbidity cases was examined by sex per thousand episodes (men 41.834 and women 15.210), twice as many women as men were diagnosed with chronic pulmonary disease, uncomplicated hypertension, and depression.

**Table 5:** **Distribution of Elixhauser comorbidity measures in HIV inpatients by gender and length of stay**

**Comorbidity**	**Frequency**	**%**	**Male (n = 41,834)**		**Female(n = 15,210)**		**LOS/SCm** ^*^	
			**%**	**‰** ^**¥**^	**%**	**‰** ^**¥**^	^**(Median)**^	**p-value** ^**#**^
Paralysis	708	1	76	13	24	11	12	<0.001
Blood loss and anemia	1,943	3	71	33	29	37	8	<0.001
Valvular heart disease	620	1	74	11	26	11	8	<0.001
Deficiency anemia	1,889	3	65	30	35	43	6	<0.001
Depression	1,597	3	77	27	23	22	6	<0.001
Weight loss	3,753	7	73	66	27	66	5	<0.001
Pulmonary circulation disorders	414	1	65	6	36	10	5	<0.001
Peptic ulcer disease excluding bleeding	205	0	45	2	55	8	5	<0.001
Hypothyroidism	148	0	37	1	64	6	5	<0.001
Other neurological disorders	3,329	6	78	60	22	46	4	<0.001
Metastatic cancer	537	1	81	10	19	7	4	<0.001
Rheumatoid arthritis/collagen vascular diseases	490	1	68	9	32	11	4	<0.001
Diabetes, complicated	397	1	65	6	35	9	4	0.008
Peripheral vascular disorders	230	0	87	5	14	2	4	<0.001
Fluid and electrolyte disorders	5,189	9	85	93	15	46	3	<0.001
Solid tumor without metastasis	2,905	5	82	57	18	34	3	<0.001
Congestive heart failure	1,029	2	73	18	27	19	3	<0.001
Cardiac arrhythmias	877	2	71	15	29	17	3	<0.001
Psychoses	616	1	71	11	29	12	3	<0.001
Liver disease	13,905	24	80	264	21	188	2	<0.001
Alcohol abuse	4,578	8	73	91	27	92	2	<0.001
Coagulopathy	3,187	6	73	58	27	59	2	<0.001
Chronic pulmonary disease	2,458	4	66	39	34	55	2	<0.001
Lymphoma	1,471	3	55	21	45	48	2	<0.001
Hypertension, uncomplicated	2,191	4	67	35	33	47	1	0.001
Diabetes, uncomplicated	1,709	3	70	29	30	34	1	0.062
Hypertension, complicated	518	1	72	9	28	9	1	0.001
Renal failure	1,929	3	66	31	34	43	0	0.425
Drug abuse	16,978	30	81	329	19	211	0	0.037
Obesity	225	0	76	4	24	3	−1	0.047

*LOS with a specific comorbidity is defined as the days above the median LOS in patients without this comorbidity. The median LOS without each specific comorbidity was 11 days for all hospitalization episodes.

^#^Statistical significance was determined by the Mann–Whitney U test with significance at p < 0.05.

^¥^Number of people in each gender per 1,000 inpatients diagnosed with a specific comorbidity.

LOS = length of hospital stay.

We also examined the number of etiologies causing the hospitalizations; the cause of hospitalization was categorized as related or not directly related to HIV. HIV-related hospitalizations decreased consistently over the study period from 82% to 75%, while hospitalizations unrelated to HIV increased from 18% to 25%. Deaths directly caused by HIV decreased from 16% to 14%, while deaths from causes unrelated to HIV increased from 9% to 12% over the decade (Additional file 1: Table S4).

In the multivariate regression analysis (adjusted and unadjusted) of HIV admissions between 2000 and 2010, we examined the variables influencing the risk of death. The risk of death increased primarily with age. Men had a 1.5 odds ratio for death compared with women. The LOS did not affect the odds ratio for death. Comorbidities, such as metastatic cancer, weight loss, fluid and electrolyte disorders, lymphoma, pulmonary circulation disorders, and cardiac arrhythmias, increased the death odds ratio more than two times compared with admissions without those comorbidities (Table 6).

**Table 6:** **Multivariate regression analyses of death odds for HIV inpatients by age, sex, LOS, and comorbidities**

**Independent variables**	**N (** ***% dead*** **)**	**OR unadjusted**	**95% C.I.**	**OR adjusted**	**95% C.I.**
**Age groups - years of age**					
1 - 0 to 17	1,057 *(2.9)*	1		1	
2 - 18 to 45	40,635 (*13.3)*	5.068	(3.541–7.253)	4.318	(2.980–6.257)
3 - 46 to 65	12,803 *(17.1)*	6.837	(4.769–9.802)	5.556	(3.826–8.069)
4 - 66 and older	2,544 *(22.9)*	9.840	(6.803–4.232)	7.955	(5.424–11.669)
**Sex**					
Female	15,210 *(11)*	1		1	
Male	41,834 *(15.6)*	1.500	(1.417–1.589)	1.407	(1.326–1.494)
**LOS median (alive/deceased)**	11.0 / 12.0	1.005	(1.005–1.006)	1.004	(1.003–1.005)
**Elixhauser comorbidity measures**					
No comorbidity*	16,850 (9.7)	1		1	
Metastatic cancer	537 *(41.5)*	4.319	(3.632–5.136)	3.286	(2.732–3.952)
Weight loss	3,753 *(30.8)*	2.917	(2.710–3.140)	2.501	(2.312–2.704)
Fluid and electrolyte disorders	5,189 *(29.9)*	2.897	(2.715–3.091)	2.332	(2.176–2.499)
Lymphoma	1,471 *(27.5)*	2.319	(2.063–2.607)	2.260	(2.000–2.553)
Pulmonary circulation disorders	414 *(22.9)*	1.782	(1.416–2.244)	2.132	(1.651–2.754)
Cardiac arrhythmias	877 *(31.1)*	2.749	(2.379–3.178)	2.030	(1.734–2.375)
Solid tumor without metastasis	2,905 *(24.1)*	1.973	(1.806–2.156)	1.706	(1.552–1.876)
Coagulopathy	3,187 *(23.8)*	1.953	(1.794–2.126)	1.668	(1.524–1.825)
Blood loss and anemia	1,943 *(22.6)*	1.780	(1.596–1.985)	1.573	(1.403–1.764)
Peripheral vascular disorders	230 *(23.9)*	1.877	(1.385–2.544)	1.416	(1.025–1.956)
Congestive heart failure	1,029 *(22.6)*	1.765	(1.522–2.046)	1.403	(1.195–1.648)
Other neurological disorders	3,329 *(20.2)*	1.551	(1.420–1.694)	1.364	(1.242–1.498)
Paralysis	708 *(21.6)*	1.654	(1.380–1.981)	1.331	(1.097–1.614)
Renal failure	1,929 *(18.9)*	1.403	(1.248–1.576)	1.221	(1.080–1.381)
Peptic ulcer disease excluding bleeding	205 *(20.5)*	1.537	(1.094–2.159)	1.208	(0.843–1.733)
Alcohol abuse	4,578 *(18.1)*	1.348	(1.245–1.459)	1.151	(1.057–1.253)
Liver disease	13,905 *(15.2)*	1.087	(1.030–1.147)	1.027	(0.969–1.088)
Hypertension, uncomplicated	2,191 *(12.7)*	0.861	(0.757–0.978)	0.685	(0.597–0.785)
Chronic pulmonary disease	2,458 *(11)*	0.726	(0.638–0.825)	0.542	(0.470–0.625)
Depression	1,597 *(9.8)*	0.642	(0.544–0.759)	0.533	(0.447–0.635)
Nutrient-deficiency anemia	1,889 *(10.7)*	0.706	(0.609–0.818)	0.531	(0.455–0.621)
Obesity	225 *(7.1)*	0.455	(0.273–0.757)	0.474	(0.281–0.799)

*Patients with each comorbidity were compared with patients without that comorbidity. Some Elixhauser comorbidities had no influence on the nonadjusted model and were not included in the analysis, namely *rheumatoid arthritis/collagen vascular diseases, psychoses, drug abuse, valvular heart disease, diabetes (both uncomplicated and complicated), complicated hypertension,* and *hypothyroidism*.

LOS = length of hospital stay; OR = odds ratio; 95% CI = 95% confidence interval.

## Discussion

The decline in HIV-related hospital admissions during the final 4 years of the study period (2007–2010) was in opposition to the trend over the first 7 years (2000–2006) in which the number of episodes increased. The HIV incidence appears to have decreased by nearly 50% in one decade. These data indicate success in controlling HIV in Portugal. This success is good considering that the worldwide HIV incidence is also decreasing (nearly 20% over the study period) [19].

Because HIV is a chronic disease, affected patients experience numerous acute exacerbations. In the present study, 80% of patients were hospitalized because of an acute illness, which highlights the challenge of clinical management and control of HIV. These acute episodes are more common in men because they form the majority of HIV patients in Portugal, as in the rest of Europe [20]. Worldwide, half of HIV patients are men, though in certain regions, such as Africa and the Caribbean, women predominate [7]. Over the study decade, the median age increased by 10 years, confirming that many PLWHIV are surviving longer [2,21,22].

The overall inpatient LOS decreased during the decade, though the rate increased during the most recent years. This trend may reflect improved access to outpatient care and better adherence to HAART [23]. Fleishman and Hellinger [3] studied trends in HIV-related inpatient admissions in seven states in the USA (1996–2000) and found a comparatively shorter LOS (median 6 days) that was half the LOS observed in this study. Characteristics unique to the Portuguese NHS may explain this difference. One reason could be that to discharge a patient in Portugal, several guarantees should be met, such as adequate family support, and continued care at home or at a center providing social assistance; these requirements likely increase the LOS. When the LOS for HIV-related admissions was compared with the LOS for all other admissions, HIV-related admissions showed a greater median LOS than all other admissions. While the LOS for HIV-related admissions decreased compared with all other admissions, this trend was not statistically significant.

Trends in mortality varied across regions, with Norte and Centro differing significantly from Lisbon, Alentejo, and Algarve. Overall, the in-hospital mortality rate decreased by 12%. In the Norte and Centro regions, the decrease between 2000 and 2010 was approximately 40% and 24%, respectively. In Lisbon and Algarve, the mortality rate increased steadily during the period; this result contrasts with the overall decreased mortality over the same period. Many mechanisms may underlie this discrepancy; the treatment methods and patient follow-up protocols are similar across the country, while the absolute frequency of admissions varies by region. The Alentejo and Algarve regions had fewer episodes; therefore, one death may have greater weight than a death in the northern regions where there are more HIV inpatient admissions. Thus, the discrepancy may be due to significant differences in the population itself, rather than in the quality of care. Our results show a substantial decrease in the mortality rate. This decrease might be due to HAART or improved monitoring of HIV patients through outpatient hospital services and health centers in the area of residence. These data confirm previously published data showing that the HIV-related mortality in Europe has decreased since 1996 [24,25].

The mean estimated cost increased from 2000 to 2001, stabilized with little fluctuation from 2001 to 2005, and decreased steadily from 2006 to 2010. There were no regional differences, and the admission complexity did not appear to vary over the period. Even considering that the prices are set by the Ministry of Health, the mean cost showed a clear decreasing trend, which may be due to the decreased number of cases or improved clinical management over the decade. However, the mean daily cost per admission increased over the same period. These results contradict the lower daily costs reported by Perelman *et al*. [26].

Regression analyses for the risk of HIV inpatient mortality were performed for age, sex, LOS, and ECI. The risk of death increased over four-fold with age, but the difference was not significant in patients older than 18 years, as indicated by the overlap in the 95% CIs. Men had a greater risk of death than women did, and the LOS did not influence the risk of mortality. Most comorbidities increased the risk of death. Metastatic cancer increased the risk three-fold. Several comorbidities did not influence the risk of death, including complicated or uncomplicated hypertension, chronic pulmonary disease, obesity, nutrient-deficiency anemia, psychosis, and depression.

This study used comprehensive discharge data compiled in mainland Portugal. Thus, the findings are more generalizable than results based on data from a single hospital. However, this study has limitations, due primarily to the nature of the data [27]. First, we cannot identify the number of unique patients, the number of times that one patient was hospitalized at a single facility, or the number of years in care. Important clinical information, mainly complementary exams (CD4 cell count, viral load) and the therapy used, was not available. To track the long-term outcomes and quality of care, an electronic database or electronic health record system specially designed to capture these important components of HIV-related care is necessary.

## Conclusions

We found a strong, consistent decrease in the hospital admission rate, cost, LOS, and mortality rate during 2000–2010. Although the rate of hospitalization directly related to HIV has decreased over time, HIV remains a major cause of hospitalization. Further scrutiny is necessary to explain this trend during the HAART era. The National Health Service appears to have persistently improved outcomes over the previous decade. However, there is an increased need for studies evaluating the constraints on hospital care of HIV/AIDS patients to determine the measures needed to improve the prevention and management of comorbidities in PLWHIV.

## Additional file

Additional file 1: Table S1. HIV-related inpatient hospitalizations in Portugal by admission type. **Table S2.** Mean daily cost (euros) per year of HIV inpatient admissions in Portugal. No. = number. **Table S3.** HIV/AIDS DRG estimated cost per year. DRG = Diagnosis-related group, as described by the Portuguese Ministry of Health. **Table S4.** Inpatient admissions and mortality according to HIV-related and -unrelated principal diagnoses. PD = principal diagnosis.
