# Supplementary material for: HIV disease burden, cost, and length of stay in Portuguese hospitals from 2000 to 2010: a cross-sectional study
Source: BMC Health Serv Res. 2015 Apr 8;15:144. doi: 10.1186/s12913-015-0801-8 (PMC4403787; doi:10.1186/s12913-015-0801-8)
Supplement: Additional file 1: Table S1. — HIV-related inpatient hospitalizations in Portugal by admission type. Table S2. Mean daily cost (euros) per year of HIV inpatient admissions in Portugal. No. = number. Table S3. HIV/AIDS DRG estimated cost per year. DRG = Diagnosis-related group, as described by the Portuguese Ministry of Health. Table S4. Inpatient admissions and mortality according to HIV-related and -unrelated principal diagnoses. PD = principal diagnosis. [file 12913_2015_801_MOESM1_ESM.doc]

**Online supplement**

Table S1. HIV-related inpatient hospitalizations in Portugal by admission type

| **Year** | **Planned** | **(%)** | **Emergency room** | **(%)** | **Recovery program for waiting lists** | **(%)** | **Total** |
| --- | --- | --- | --- | --- | --- | --- | --- |
| **2000** | 757 | (14) | 4,702 | (86) | 0 | (0.0) | 5,459 |
| **2001** | 831 | (15) | 4,719 | (85) | 0 | (0.0) | 5,550 |
| **2002** | 1,124 | (20) | 4,530 | (80) | 0 | (0.0) | 5,654 |
| **2003** | 1,174 | (21) | 4,447 | (79) | 7 | (0.1) | 5,628 |
| **2004** | 1,238 | (23) | 4,227 | (77) | 3 | (0.1) | 5,468 |
| **2005** | 963 | (18) | 4,523 | (82) | 5 | (0.1) | 5,491 |
| **2006** | 864 | (17) | 4,334 | (83) | 16 | (0.3) | 5,214 |
| **2007** | 778 | (15) | 4,296 | (84) | 15 | (0.3) | 5,089 |
| **2008** | 807 | (17) | 4,077 | (83) | 10 | (0.2) | 4,894 |
| **2009** | 713 | (17) | 3,607 | (83) | 12 | (0.3) | 4,332 |
| **2010** | 686 | (16) | 3,573 | (84) | 7 | (0.2) | 4,266 |
| **Total** | 9,935 | (17) | 47,035 | (83) | 75 | (0.1) | 57,045 |

Table S2. Mean daily cost (euros) per year of HIV inpatient admissions in Portugal

| Year | No. of episodes | Average daily cost per year | Total estimated cost per year |
| --- | --- | --- | --- |
| 2000 | 4060 | 248.65 € | **19,278,995.08 €** |
| 2001 | 4114 | 248.17 € | **20,103,602.66 €** |
| 2002 | 4210 | 243.61 € | **20,489,384.65 €** |
| 2003 | 4109 | 252.04 € | **20,410,455.46 €** |
| 2004 | 4029 | 252.59 € | **20,318,573.31 €** |
| 2005 | 4155 | 247.74 € | **20,639,255.83 €** |
| 2006 | 4074 | 263.32 € | **20,008,134.96 €** |
| 2007 | 3885 | 259.21 € | **18,385,974.97 €** |
| 2008 | 3675 | 263.03 € | **17,989,726.68 €** |
| 2009 | 3270 | 259.24 € | **15,890,571.34 €** |
| 2010 | 3196 | 260.15 € | **15,124,535.55 €** |
| Total | 42,777 | 253.78 € | **208,639,210.49 €** |

No. = number

Table S3. HIV/AIDS DRG estimated cost per year

| GDH_AP21 | No. of episodes | Annual cost of DRGs | % |
| --- | --- | --- | --- |
| 700 | 106 | 4,246,667.40 € | 2.0 |
| 701 | 241 | 3,566,848.20 € | 1.7 |
| 702 | 255 | 3,070,679.40 € | 1.5 |
| 703 | 1361 | 14,527,123.46 € | 7.0 |
| 704 | 1206 | 7,692,567.48 € | 3.7 |
| 705 | 1973 | 17,635,640.77 € | 8.5 |
| 706 | 1801 | 15,967,900.13 € | 7.7 |
| 707 | 1699 | 14,637,292.76 € | 7.0 |
| 708 | 910 | 7,747,412.40 € | 3.7 |
| 710 | 7108 | 42,879,436.48 € | 20.6 |
| 711 | 2685 | 13,818,808.95 € | 6.6 |
| 712 | 3397 | 11,388,748.23 € | 5.5 |
| 713 | 808 | 1,527,443.20 € | 0.7 |
| 714 | 12,902 | 39,338,068.98 € | 18.9 |
| 715 | 2768 | 4,886,405.76 € | 2.3 |
| 716 | 3557 | 5,708,166.89 € | 2.7 |
| Total | 42777 | **208,639,210.49 €** | 100.0 |

DRG = Diagnosis-related group, as described by the Portuguese Ministry of Health

Table S4. Inpatient admissions and mortality according to HIV-related and -unrelated principal diagnoses

|  |  | **HIV/AIDS - PD** | **HIV/AIDS death - PD** |
| --- | --- | --- | --- |
| **Year** | **Patient** | **related/not related** | **related/not related** |
|  | no. | (%) | No. ***(%)*** |
| 2000 | 5459 | 82/18 | 726 ***(16.2)***/86 ***(8.7)*** |
| 2001 | 5550 | 83/17 | 777 ***(17)***/85 ***(8.6)*** |
| 2002 | 5654 | 82/18 | 775 ***(16.8)***/94 ***(9.1)*** |
| 2003 | 5628 | 80/20 | 758 ***(16.8)***/93 ***(8.4)*** |
| 2004 | 5468 | 80/20 | 724 ***(16.7)***/92 ***(8.2)*** |
| 2005 | 5491 | 80/20 | 730 ***(16.7)***/84 ***(7.6)*** |
| 2006 | 5214 | 83/17 | 644 ***(14.9)***/73 ***(8.2)*** |
| 2007 | 5089 | 79/21 | 604 ***(15)***/79 ***(7.5)*** |
| 2008 | 4894 | 78/22 | 537 ***(14)***/103 ***(9.8)*** |
| 2009 | 4332 | 77/23 | 513 ***(15.4)***/66 ***(6.6)*** |
| 2010 | 4266 | 75/25 | 435 ***(13.6)***/125 ***(11.7)*** |
| Total | 57,045 | 80/20 | 7223 ***(15.8)***/980 ***(8.6)*** |

PD = principal diagnosis
